# Circulating microRNAs as Early Biomarkers of Breast Cancer: A Nested Case-Control Study Within a Prospective Cohort in Italy

**DOI:** 10.3390/ijms27062706

**Published:** 2026-03-16

**Authors:** Lisa Padroni, Giorgia Marmiroli, Laura De Marco, Valentina Fiano, Saverio Caini, Claudia Agnoli, Claudia Vener, Vittorio Simeon, Salvatore Panico, Luca Manfredi, Lorenzo Milani, Fulvio Ricceri, Carlotta Sacerdote

**Affiliations:** 1 Department of Clinical and Biological Sciences, Centre for Biostatistics, Epidemiology, and Public Health (C-BEPH), University of Turin, 10043 Orbassano, Italy; lisa.padroni@unito.it (L.P.); luca.manfredi@unito.it (L.M.);; 2 Unit of Cancer Epidemiology, Department of Medical Sciences, University of Turin, 10126 Turin, Italyvalentina.fiano@unito.it (V.F.); 3 Unit of Cancer Epidemiology, Città Della Salute e Della Scienza University-Hospital and Center for Cancer Prevention (CPO), Via Santena 7, 10126 Turin, Italy; 4 Cancer Risk Factors and Lifestyle Epidemiology Unit, Institute for Cancer Research, Prevention and Clinical Network (ISPRO), 50139 Florence, Italy; 5 Epidemiology and Prevention Unit, Department of Epidemiology and Data Science, Fondazione IRCCS Istituto Nazionale dei Tumori, 20133 Milan, Italy; claudia.agnoli@istitutotumori.mi.it (C.A.); claudia.vener@istitutotumori.mi.it (C.V.); 6 Medical Statistics Unit, University of Campania “Luigi Vanvitelli”, 80138 Napoli, Italy; vittorio.simeon@unicampania.it; 7 Department of Clinical Medicine and Surgery, School of Medicine, Federico II University, 80138 Naples, Italy; 8 Department of Health Sciences, University of Eastern Piedmont, Via Solaroli 17, 28100 Novara, Italy; 9 Unit of Epidemiology, Local Health Unit of Novara, Viale Roma, 7, 28100 Novara, Italy

**Keywords:** breast cancer, circulating microRNAs, miR-181, Let7, droplet digital PCR, nested case–control

## Abstract

Circulating microRNAs (miRNAs) are promising minimally invasive biomarkers for cancer risk assessment, yet prospective evidence for breast cancer (BC) remains limited. We conducted a nested case–control study within a prospective cohort to examine whether pre-diagnostic circulating miRNAs are associated with subsequent BC risk and to explore their potential relevance in prospective population-based settings. Baseline serum from 160 women (80 incident BC cases; 80 matched controls) was analyzed, with a median time to diagnosis of 8.9 years. Eight candidate miRNAs were quantified by droplet digital PCR (ddPCR) and normalized to miR-484. Group differences were evaluated by non-parametric tests, and odds ratios for BC were estimated using logistic regression models adjusted for established risk factors, with Bonferroni correction for multiple testing. Cases and controls were comparable at baseline. Among the candidates, lower circulating miR-181 levels showed a suggestive inverse association with BC risk in fully adjusted models, while lower Let7 levels showed only a non-significant, hypothesis-generating inverse trend that did not survive Bonferroni correction. No other miRNA displayed clear associations with BC risk. These findings, while preliminary, support further large-scale prospective investigations specifically designed to assess predictive performance and external validation. employing standardized pre-analytical and analytical protocols, repeated sampling, and independent replication/external validation to clarify the etiologic relevance and potential risk-prediction value of circulating miRNAs for BC.

## 1. Introduction

Breast cancer (BC) is one of the leading causes of cancer-related morbidity and mortality worldwide, highlighting how crucial early diagnosis is for improving patient outcomes [1]. Molecular biomarkers found in body fluids show great promise for non-invasive early detection and tailored patient management [2]. Among these, circulating microRNAs (miRNAs) have been recognized as reliable indicators of disease presence or progression, easily detectable in serum and plasma because of their stability against RNase degradation, although their precise role in cancer initiation and progression remains controversial [3,4]. Since the pilot study by Zhao et al. [5], which recognized differentially ex-pressed panels of circulating miRNAs that set early-stage BC patients apart from healthy individuals, extensive research has attempted to replicate and extend these results [6]. However, most published studies to date have been cross-sectional or case-control analyses in patients with established BC, raising concerns about reverse causality and the validity of the associations for true risk prediction [7].

Prospective cohort studies with a nested case–control design provide a powerful framework to overcome these limitations. By using pre-diagnostic blood samples collected before disease onset, they allow researchers to investigate whether alterations in circulating miRNA levels precede cancer development and can therefore serve as predictive biomarkers [7]. Despite the theoretical advantages, relatively few nested case–control studies have investigated circulating miRNAs in relation to incident BC risk. For example, a nested case–control analysis within the Sister Study reported 21 serum miRNAs differentially expressed between future BC cases and controls, and in a small independent set it verified overexpression of the three top signals, miR-18a, miR-181a, and miR-222, although not at statistical significance [8]. A nested case–control study within the Italian ORDET cohort identified 20 leukocyte miRNAs differentially expressed between postmenopausal women who developed BC and matched controls; in particular, low baseline levels of miR-145-3p (OR = 2.16, 95% CI: 1.20–3.90, *p* = 0.01) and miR-145-5p (OR = 2.02, 95% CI: 1.15–3.54, *p* = 0.02) were significantly associated with increased long-term BC risk [9]. Similarly, a nested analysis within the ANDROMEDA breast cancer screening cohort found that a panel of seven pre-diagnostic plasma miRNA ratios, such as miR-199a-3p/Let-7a-5p and miR-21-5p/miR-23a-3p, combined with lifestyle and clinical factors, predicted BC occurrence with good discrimination (AUC = 0.79; sensitivity = 71.9%, specificity = 76.6%) [10].

Finally, a prospective analysis within the Framingham Heart Study identified two plasma extracellular miRNAs, miR-134-5p and miR-505-3p, significantly associated with incident BC over a median follow-up of 15.7 years (HR = 0.88, 95% CI: 0.81–0.96, *p* = 0.002 for miR-134-5p; HR = 0.85, 95% CI: 0.76–0.95, *p* = 0.005 for miR-505-3p) [11]. These examples illustrate that well-designed prospective studies are essential to identify and refine putative associations between circulating miRNAs and outcomes and to evaluate their potential utility in population-based contexts, however, they also underscore the urgent need for independent replication and external validation of differentially expressed miRNAs reported in previous investigations.

Motivated by these considerations, we aim to examine the association between a predefined panel of circulating miRNAs and the subsequent development of BC. We specifically evaluate miRNA expression profiles measured several years prior to the clinical onset of the disease, using a nested case-control design within a large prospective cohort. For the present analysis, we selected eight circulating miRNAs: Let7, miR-21, miR-155, miR-181, miR-222, miR-145, miR-92a, and miR-20a. These candidates were chosen on the basis of previous evidence linking them to BC pathogenesis across different stages of tumor development, from initiation and progression to metastatic spread, and all have also been implicated in inflammatory pathways that play a central role in tumor biology [6,12]. Notably, we applied the same analytical framework in a previous investigation conducted by our group, in which cases and controls were drawn from the same cohort and the identical set of miRNAs was examined in relation to colorectal cancer risk [13].

## 2. Results

The study included 160 participants, 80 newly diagnosed cases of BC and 80 healthy controls. Among the cases, the median follow-up time to diagnosis was 8.9 years, with a range from 0.39 to 14.61 years. All analyzed socio-demographic characteristics were similar between cases and controls. No significant difference was found in the mean age at recruitment between cases and controls (50.5 vs. 50.0 years; *p* = 0.80), nor regarding smoking status, body mass index, levels of physical activity, adherence to the Mediterranean diet, or socioeconomic position measured by the Relative Index of Inequality (see Table 1).

**Figure 1:**
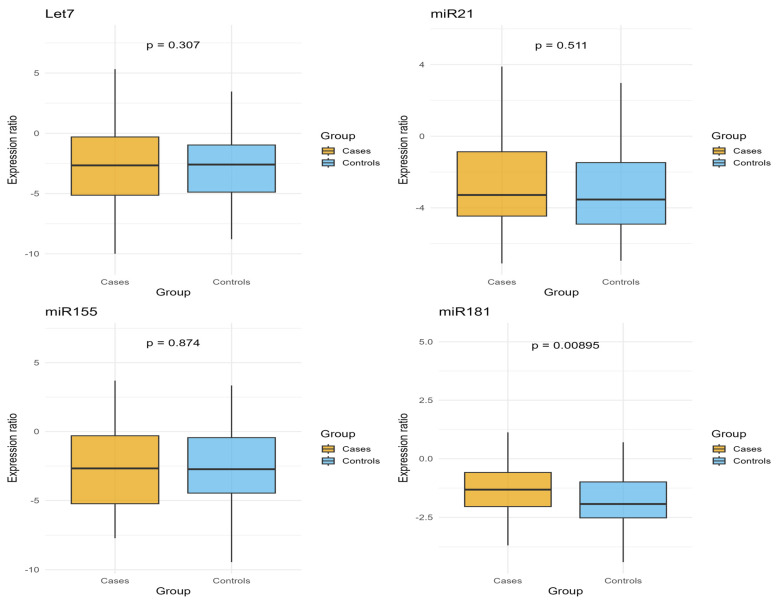

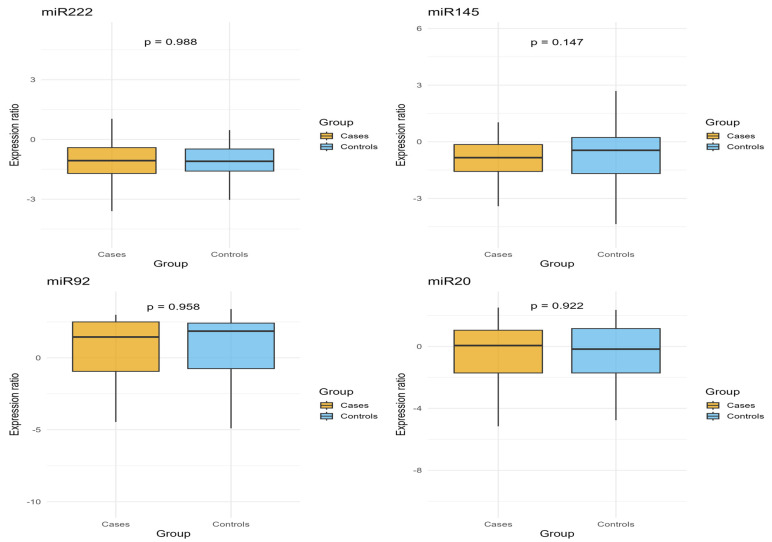
Expression levels of normalized and log-transformed selected miRNAs (Let7, miR-21, miR-155, miR-181, miR-222, miR-145, miR-92, and miR-20) in incident BC cases and healthy controls. Box plots display the median values (horizontal line) with interquartile ranges (IQR; boxes) and whiskers indicating the data range. *p*-values are from Wilcoxon rank-sum tests comparing the distribution of expression levels between cases and controls for each miRNA.

The expression levels of eight miRNAs (Let7, miR-21, miR-155, miR-181, miR-222, miR-145, miR-92, and miR-20) were evaluated. Among these, only miR-181 showed a statistically significant difference in expression between cases and controls (*p* = 0.009). The remaining miRNAs did not show any significant differences (Figure 1). Overall, individual serum miRNA levels showed substantial between-subject variability.

Comparing the average expression levels of the eight selected miRNAs between incident BC cases and healthy controls, four miRNAs, Let7, miR-21, miR-181, and miR-92, showed higher expression in the controls, while miR-155, miR-222, miR-145, and miR-20 showed higher average expression in the cases. The fold change values, calculated as the ratio of average expression in cases to controls, ranged from 0.28 for Let7 to 1.80 for miR-155 (Table 2). In particular, Let7 showed the greatest downregulation in cases compared to controls, with a fold change of 0.28, while miR-155 demonstrated the greatest upregulation, with a fold change of 1.80.

Univariate conditioned logistic regression analyses were conducted to examine the association between circulating levels of each miRNA and BC risk. Only one miRNA, miR-181, showed a significant inverse association, with an odds ratio (OR = 0.59; 95% CI: 0.41–0.85; *p* = 0.036), suggesting that increased expression of miR-181 is associated with a risk reduction of 30% of developing BC; the result remained statistically significant after adjustment for multiple comparisons (Bonferroni). All other miRNAs showed no significant association with BC risk, confirming Wilcoxon test results (Table 3).

A multivariate conditioned logistic regression analysis was performed to evaluate the association between the expression of the eight selected miRNAs and BC risk, adjusting for confounding factors including age at recruitment, smoking status, BMI, physical activity, adherence to the Mediterranean diet and socioeconomic position as measured by the relative inequality index (RII). In this model the miR-181 showed an inverse association with BC, with an odds ratio (OR = 0.55; 95% CI: 0.36–0.84, *p* = 0.006); also, this result remained statistically significant after adjustment for multiple comparisons using Bonferroni correction (Table 4). In the fully adjusted model, Let7 also reached nominal statistical significance (ORadj = 0.81; 95% CI: 0.67–0.98; *p* = 0.030), with higher circulating levels associated with lower odds of BC (Table 4). However, in both the univariate and multivariable models the association for Let7 did not remain statistically significant once Bonferroni correction for multiple comparisons was applied, and it should therefore be regarded as suggestive rather than conclusive (Table 3 and Table 4).

## 3. Discussion

Circulating microRNAs (miRNAs) have emerged as promising non-invasive biomarkers for early cancer detection, including BC, based on their stability in peripheral blood and their functional role in tumorigenesis [14,15,16]. Our nested case–control study within the EPIC-Italy cohort contributes to this growing body of literature by examining the association between eight circulating miRNAs and the future risk of BC using pre-diagnostic serum samples. Among the selected candidates, only miR-181 showed a significant inverse association with BC incidence, suggesting a potential protective role.

The inverse association between circulating miR-181 and breast cancer risk observed in our study is biologically plausible but must be interpreted within the complex, compartment-specific biology of the miR-181 family. miR-181a/b regulates DNA damage response pathways, including attenuation of ATM signaling, and influence genomic stability in breast epithelial cells, where they have been reported to exert context-dependent oncogenic effects [17]. At the same time, miR-181 plays a central role in immune regulation, modulating T-cell receptor sensitivity and thymic selection, thereby shaping peripheral immune responsiveness [18].

This dual functionality may help reconcile discrepancies across prospective studies (Table S1). In the Sister Study, higher pre-diagnostic serum miR-181a levels were observed among future cases, although the association was not statistically significant after validation [8]. In contrast, case–control studies have reported reduced circulating miR-181a in women with established disease [19]. Such heterogeneity likely reflects the fact that circulating miRNAs do not directly mirror tumor-cell expression but rather represent an integrated systemic signal derived from epithelial, stromal, and immune compartments. In a pre-diagnostic context, years before clinical detection, circulating miR-181 may predominantly reflect host immune and genomic stability pathways rather than tumor burden.

Reduced systemic miR-181 levels could therefore indicate impaired immune surveillance and diminished regulation of DNA damage responses, conditions permissive to early tumor initiation. This interpretation is consistent with the distribution of tumors in our cohort, where more than 80% were ER-positive. Luminal tumors are characterized by proliferative signaling and reliance on DNA damage response pathways, including ATM-mediated mechanisms, as well as sensitivity to immune–inflammatory modulation. Thus, lower circulating miR-181 may mark a systemic biological environment favoring luminal tumor development rather than functioning as a direct tumor-suppressive signal per se.

Overall, our findings support the view that pre-diagnostic circulating miR-181 captures aspects of host systemic biology relevant to breast carcinogenesis, underscoring the importance of distinguishing circulating biomarker dynamics from tumor-intrinsic expression patterns.

Regarding Let7, converging preclinical evidence supports a tumor-suppressor role in breast carcinogenesis, with reduced Let7 promoting self-renewal and tumorigenicity of breast tumor-initiating cells [20]. Mechanistically, Let7 directly represses key oncogenic drivers such as RAS and HMGA2, and disruption of the Let7–HMGA2 interaction enhances oncogenic transformation [21,22]. In breast tumour-initiating cells, downregulation of Let7 sustains self-renewal, sphere-forming ability and metastatic potential, whereas enforced Let7 expression reduces clonogenicity and tumour growth in vivo, in part through repression of RAS, HMGA2 and other components of epithelial–mesenchymal transition and stemness programmes [20,21,22]. Let7 family members have also been implicated in the modulation of hormone receptor–related signalling pathways, including oestrogen and androgen receptor axes, providing an additional link between Let7 dysregulation and more aggressive or hormone-independent breast cancer phenotypes. In our fully adjusted model, higher circulating Let7 levels were associated with lower odds of BC, although the signal was not evident in univariate analyses and did not survive Bonferroni correction, so this finding should be considered hypothesis-generating. At the clinical level, case–control evidence reports lower serum Let7 in women with breast cancer compared with healthy controls (90 cases vs. 64 controls), supporting a possible inverse relationship in circulation [23]. Diagnostic panels that include Let7a have also shown utility in distinguishing cases from controls and, importantly, Let7a levels tend to decrease to near-control values after curative surgery, suggesting that part of the circulating signal reflects tumor burden rather than antecedent risk [24]. Consistently, longitudinal monitoring studies have reported post-treatment increases in circulating Let7a, aligning with a disease-burden interpretation [25,26]. Importantly, none of the other six miRNAs evaluated showed significant associations with breast cancer risk. Although limited statistical power cannot be excluded, this pattern suggests relative biological specificity for miR-181, and possibly Let7, within this dataset. Similar observations have been reported in other prospective cohorts (e.g., ESTHER, Rotterdam), where only a subset of circulating miRNAs demonstrated reproducible associations, often varying by cancer type, latency interval, and analytical platform [15,16].

Collectively, these findings underscore the need for large, methodologically harmonized prospective studies. Pre-analytical variability, normalization strategies, and platform differences remain major sources of heterogeneity in circulating miRNA research and likely contribute to inconsistent replication [27].

Beyond etiologic insight, the translational relevance of circulating miRNAs will depend on their integration into multivariable prediction models alongside established risk factors and clinical biomarkers. Traditional serum markers such as CA125 and CA15.3 show limited performance for early detection [28], and composite models incorporating miRNA signatures may improve discrimination [29]. However, given the present sample size, we did not construct multi-miRNA prediction models or formally assess incremental predictive performance beyond conventional markers, as such analyses would not permit stable internal validation.

Finally, circulating miRNA levels may vary over time. Prospective studies incorporating repeated sampling will be essential to characterize temporal dynamics and strengthen causal interpretation of pre-diagnostic associations [30].

The main strengths of our study include its prospective design, the use of pre-diagnostic serum samples, and a matching strategy that minimizes confounding. The median follow-up of nearly nine years enhances our ability to detect long-term predictive associations. Limitations include limited statistical power for modest effects and subtype-stratified analyses, reliance on a single time-point measurement, and inability to explore time-to-diagnosis heterogeneity. The study was powered to detect relatively large effect sizes, and non-significant associations for other miRNAs may reflect limited statistical power rather than absence of biological effect. Therefore, potential type II error cannot be excluded for null findings.

We did not attempt to derive or report diagnostic performance metrics (such as AUC, sensitivity, and specificity) for individual miRNAs or to propose specific cut-off values. In the absence of standardized pre-analytical and analytical procedures for circulating miRNAs (including extraction protocols, detection platforms, normalization strategies, and reference miRNAs), any threshold identified in this study would be assay and protocolspecific and would have limited generalizability. Robust evaluation of diagnostic performance will require standardized methodologies and the formal development, internal validation, and external validation of multi-marker models that integrate miRNAs with traditional risk factors and clinical biomarkers in larger, independent datasets. In fact, we used miR-484 as endogenous reference based on prior reports of its relative stability in circulation; however, we did not perform a dedicated pre-analytical stability sub-study within our dataset, nor did we include an exogenous spike-in control at the RNA extraction step to directly estimate extraction efficiency. Although ddPCR reduces variability related to amplification efficiency, residual technical variability related to RNA recovery cannot be entirely excluded. Importantly, given the prospective design and identical handling of case and control samples, any non-differential degradation or extraction variability would likely bias associations toward the null rather than generate spurious inverse associations.

In conclusion, our findings suggest that higher circulating levels of miR-181 and probably Let7, may be associated with reduced BC risk, providing preliminary evidence for their role as a potential biomarkers for early detection. This observation complements prior research from the Sister Study [8], Framingham cohort [11], and other European cohorts [10,16], emphasizing the promise of circulating miRNAs in cancer risk prediction. Validation in larger, independent cohorts and mechanistic studies are needed to clarify the biological role of miR-181 and Let7 and assess their clinical utility.

## 4. Materials and Methods

### 4.1. Study Design and Population

This investigation used a nested case-control approach within the EPIC-Italy cohort, focused on participants from the Varese and Naples centers. EPIC-Italy is a segment of the European Prospective Investigation into Cancer and Nutrition (EPIC) study, which is a large-scale, multicenter prospective cohort with over 500,000 individuals from ten European nations [31]. In Italy, around 47,000 adults were enrolled between 1993 and 1998 across five centers, including Florence, Turin, Naples, Ragusa, and Varese. Upon enrollment, participants completed comprehensive standardized questionnaires taking information on dietary habits and lifestyle factors, meantime physical measurements and blood collection were collected. Blood samples were obtained at baseline, well in advance of any subsequent cancer diagnoses, enabling forward-looking analyses of biomarkers.

### 4.2. Case and Control Selection

All new BC cases diagnosed after participant enrollment were identified through cancer registry data and active follow-up procedures, with monitoring continuing until 2010. Individuals with a prior history of cancer were excluded from the study. Only cases confirmed histologically as malignant adenocarcinoma of the breast were included. From each center, 65 cases were chosen and matched individually to healthy control subjects through incidence density sampling. Matching factors comprised age at enrollment (within ±2.5 years), recruitment center, date of enrollment (within ±6 months), and hormone replacement therapy usage. Inclusion required complete baseline information from dietary and lifestyle questionnaires.

### 4.3. Selection of miRNAs

The microRNAs examined, Let7, miR-21, miR-155, miR-181, miR-222, miR-145, miR-92, and miR-20, were selected based on a comprehensive review of the literature [12]. Priority was given to miRNAs demonstrating the most significant associations with case-control status in population studies of Caucasian populations, where expression levels were measured in serum samples.

### 4.4. Laboratory Methods

Serum samples were obtained from the EPIC-Italy cohort, where venous blood was collected at baseline and processed on the day of collection; serum was separated, aliquoted, and stored in liquid nitrogen containers according to standardized EPIC protocols for blood collection, processing, and long-term storage [30].

Total RNA was extracted from serum of selected patients using the miRNeasy Serum/Plasma Advanced Kit (Qiagen, Hilden, Germany) according to the manufacturer’s protocol and stored at −80 °C.

Reverse transcription was carried out using the miRCURY LNA RT Kit (Qiagen) with the following modification to the manufacturer’s protocol: 3 ul of RT SYBR Green Reaction Buffer, 1.5 μL of miRCURY RT Enzyme Mix, 0.75 μL of UniSp6 RNA Spike, 3 μL of RNA sample, and 6.75 μL of H_2_O reaching a final volume of 15 μL. The thermal cycling was conducted according to the manufacturer’s instructions.

QX200 Droplet Digital PCR System (Biorad, Laboratories, Hercules, CA, USA) was used to measure the expression level of miR-21, miR-155, miR-222, miR-145, miR-181, miR-92, and Let7. A PCR mix with a final volume of 21.5 μL was prepared containing 10 μL of QX200 EvaGreen SuperMix (Biorad), 2 μL of commercial miRCURY LNA miRNA PCR Assay (Qiagen, Hilden, Germany) specific for each selected miRNA. These are proprietary, pre-validated primer assays supplied by the manufacturer, 8 μL of di H_2_O and 1.5 μL of di cDNA sample. Thermal cycling conditions were as follows: 95 °C for 5 min, then 40 cycles of 95 °C for 30 s and 58 °C for 1 min, and three final steps at 4 °C for 5 min, 90 °C for 5 min, and a 4 °C infinite hold.

Data analysis was performed using the QX Manager Software 2.0 Standard Edition, and the results were considered valid if the number of counted droplets reached at least 10,000. The miRNA concentration is calculated as miRNA copies/μL normalized to miR-484. Circulating miRNA concentrations were normalized to miR-484, selected as endogenous reference based on prior evidence of its relative stability in human serum and plasma across different pathological conditions and population-based settings. In the context of droplet digital PCR (ddPCR), which provides absolute quantification and is less dependent on amplification efficiency compared with qPCR-based approaches, normalization to a stable endogenous miRNA was considered appropriate to account for inter-sample variability in input RNA and technical handling. As a technical quality control, an exogenous spike-in RNA (UniSp6, Qiagen) was added during reverse transcription to monitor RT efficiency and batch consistency. However, no exogenous spike-in was added prior to RNA extraction to formally quantify extraction efficiency. All serum samples were processed according to standardized EPIC protocols and stored in liquid nitrogen since baseline collection, minimizing degradation and avoiding repeated freeze–thaw cycles.

### 4.5. Statistical Analysis

A descriptive analysis was conducted to examine miRNA expression levels in cases and controls by calculating the mean normalized expression values for each miRNA and determining the fold change between the two groups. The Wilcoxon rank-sum test was used to evaluate differences in expression distributions for each miRNA, with results displayed through boxplots. To correct for multiple hypothesis testing, *p*-values were adjusted using the Bonferroni method.

Univariate conditioned logistic regression models were applied individually for each miRNA to estimate the unadjusted association between miRNA levels and case-control status. This was followed by multivariate conditioned logistic regression analyses. Multivariate analyses were conducted using conditional logistic regression, which accounts for the case-control matching by including the matching stratum in the model. For each miRNA, an adjusted model was estimated by including potential confounding factors selected a priori (BMI, smoking, physical activity, adherence to the Mediterranean diet, and socioeconomic status) as covariates, in order to obtain estimates of the association independent of these variables. For every miRNA, odds ratios with 95% confidence intervals and *p*-values were reported.

BMI was classified into three categories: normal weight (<25), overweight (25–30), and obese (>30). Age was included as a continuous variable. Smoking status was categorized as current smokers, former smokers, or never smokers. Physical activity was quantified by a validated index that includes occupational, household, and leisure activities, expressed in metabolic equivalent task-hours per week (MET-h/week). Participants were stratified into four groups—inactive, moderately inactive, moderately active, and active—based on sex-specific quartiles of this index.

Adherence to the Mediterranean diet was assessed using the Mediterranean Diet Score (MDS), which evaluates intake of nine dietary components: vegetables, legumes, fruits and nuts, cereals, fish, meat, dairy, alcohol, and the ratio of monounsaturated to saturated fats. Each component was scored 0 or 1 according to sex-specific median consumption, yielding a total score from 0 (lowest adherence) to 9 (highest adherence). The MDS has been validated as an effective measure of Mediterranean diet adherence within the EPIC cohort.

Socioeconomic position was captured by the Relative Index of Inequality (RII), which integrates sex, age group, and education level into a composite measure reflecting relative socioeconomic status. The RII was divided into three groups: high (RII = 1), intermediate (RII = 2), and low (RII = 3), where lower values indicate higher socioeconomic standing.

All analyses were performed using R Studio (version 4.4.3).

## Figures and Tables

**Table 1:** Baseline socio-demographic characteristics of the study population, including 160 participants (80 incident BC cases and 80 healthy controls). Values are presented as mean ± standard deviation (SD) or number (percentage). *p*-values are based on the Wilcoxon rank sum test, Pearson’s Chi-squared test, or Fisher’s exact test, as appropriate.

Characteristics	Cases *n* = 80	Controls *n* = 80	*p*-Value
**Age (mean ± SD)**	50.5 ± 8.0	50.1 ± 8.0	0.80
**Center**			0.90
Florence	25 (31%)	25 (31%)	
Turin	55 (69%)	55 (69%)	
**Smoking Status**			0.90
Never smokers	43 (54%)	43 (54%)	
Former smokers	15 (19%)	17 (21%)	
Smokers	22 (28%)	20 (25%)	
**Body Mass Index**			0.40
Normal weight (BMI < 25)	35 (44%)	43 (54%)	
Overweight (25 < BMI < 30)	33 (41%)	29 (36%)	
Obese (BMI > 30)	12 (15%)	8 (10%)	
**Physical Activity**			0.40
Active	2 (2.5%)	5 (6.3%)	
Moderately active	39 (49%)	39 (49%)	
Moderately inactive	24 (30%)	27 (34%)	
Inactive	15 (19%)	9 (11%)	
**Mediterranean Score**			0.90
0–2	16 (20%)	15 (19%)	
3–5	45 (56%)	44 (55%)	
6–9	19 (24%)	21 (26%)	
**Socioeconomic Position**			0.80
High	26 (33%)	29 (36%)	
Medium	29 (36%)	25 (31%)	
Low	25 (31%)	26 (33%)	

Physical activity was assessed using the EPIC physical activity index based on occupational and leisure activities and categorized into four levels (inactive, moderately inactive, moderately active, active) according to sex-specific quartiles. Socioeconomic position was assessed using the Relative Index of Inequality (RII), a composite measure based on education, age group, and sex, and categorized into three levels (high, intermediate, and low).

**Table 2:** Fold change in cases and controls for the panel of eight selected miRNAs. Mean expression levels were calculated for incident BC cases and healthy controls, and fold change was computed as the ratio of mean expression in cases to mean expression in controls.

miRNA	Mean Expression in Cases	Mean Expression in Controls	Fold Change
Let7	0.94	3.38	0.28
miR-21	0.91	1.26	0.72
miR-155	1.73	0.96	1.80
miR-181	0.43	0.92	0.46
miR-222	0.96	0.63	1.52
miR-145	1.28	1.23	1.05
miR-92	3.29	3.42	0.96
miR-20	1.38	1.34	1.03

**Table 3:** Univariate logistic regression odds ratios, 95% confidence intervals, and corresponding *p*-values for the association between circulating levels of eight selected miRNAs and the risk of incident BC. *p*-values adjusted for multiple comparisons were calculated using the Bonferroni correction.

miRNA	OR	Low IC 95%	High IC 95%	*p*-Value	Bonferroni Adjusted *p*-Value
Let7	0.89	0.77	1.02	0.095	0.760
miR-21	0.92	0.78	1.09	0.337	1.000
miR-155	1.01	0.88	1.17	0.865	1.000
miR-181	0.59	0.41	0.85	0.005	0.036
miR-222	0.94	0.70	1.28	0.709	1.000
miR-145	0.89	0.71	1.10	0.268	1.000
miR-92	1.00	0.87	1.14	0.952	1.000
miR-20	0.95	0.80	1.14	0.601	1.000

**Table 4:** Multivariate logistic regression odds ratios, 95% confidence intervals, and corresponding *p*-values for the association between circulating levels of eight selected miRNAs and the risk of incident BC. *p*-values adjusted for multiple comparisons were calculated using the Bonferroni correction.

miRNA	OR	Low IC 95%	High IC 95%	*p*-Value	Bonferroni Adjusted *p*-Value
Let7	0.81	0.67	0.98	0.030	0.242
miR-21	0.87	0.71	1.05	0.152	1.000
miR-155	1.02	0.86	1.20	0.825	1.000
miR-181	0.55	0.36	0.84	0.006	0.048
miR-222	0.87	0.60	1.26	0.478	1.000
miR-145	0.89	0.71	1.12	0.321	1.000
miR-92	1.00	0.00	6.02	0.253	1.000
miR-20	0.94	0.76	1.16	0.544	1.000

## Data Availability

The original contributions presented in this study are included in the article/Supplementary Material. Further inquiries can be directed to the corresponding authors.

## Supplementary Materials

The supporting information can be downloaded at https://www.mdpi.com/article/10.3390/ijms27062706/s1.

## Abbreviations

AUC—Area Under the Curve, ATM—Ataxia Telangiectasia Mutated, BC—Breast Cancer, BMI—Body Mass Index, CA125—Cancer Antigen 125 CA15.3—Cancer Antigen 15-3, CI—Confidence Interval, ddPCR—Droplet Digital Polymerase Chain Reaction, DNA—Deoxyribonucleic Acid, EPIC—European Prospective Investigation into Cancer and Nutrition, ER—Estrogen Receptor, HR—Hazard Ratio, HRT—Hormone Replacement Therapy, IQR—Interquartile Range, LNA—Locked Nucleic Acid, MDS—Mediterranean Diet Score, MET—Metabolic Equivalent of Task, miRNA—MicroRNA, OR—Odds Ratio, PCR—Polymerase Chain Reaction, RII—Relative Index of Inequality, RNA—Ribonucleic Acid, RT—Reverse Transcription, SD—Standard Deviation.
