# Supplementary material for: Circulating microRNAs as Early Biomarkers of Breast Cancer: A Nested Case-Control Study Within a Prospective Cohort in Italy
Source: Int J Mol Sci. 2026 Mar 16;27(6):2706. doi: 10.3390/ijms27062706 (PMC13026274; doi:10.3390/ijms27062706)
Supplement: Supplementary file 1 [file ijms-27-02706-s001.zip › ijms-4137872-supplementary.pdf]

Table S1. Comparison of prospective studies evaluating circulating or blood-derived miRNAs and subsequent breast cancer risk.

Cohort (Country) – Author & Year	Design & Sample Size	Platform / Normalization	Main signals reported	miR-181 / miR-222 / miR-145 (Direction vs. Cases)
EPIC (Italy) – Padroni et al., 2026	Nested case–control; 80 cases / 80 controls	ddPCR; normalization to miR-484; Bonferroni correction applied	miR-181 inversely associated with risk (significant after Bonferroni). Let-7 signal, not significant after correction. Other panel miRNAs: null (miR-21, miR-155, miR-222, miR-145, miR-92, and miR-20).	miR-181: lower in cases (higher levels associated with reduced risk). miR-222: null. miR-145: null.
Sister Study (USA) – Godfrey et al., 2013	Nested case–control; 205 cases / 205 controls	Affymetrix microarray (screening) + qRT-PCR validation; no multiple-testing correction	21 miRNAs differentially expressed (small fold-changes). miR-18a, miR-181a, miR-222 higher in cases in validation subset.	miR-181a: higher in cases. miR-222: higher in cases. miR-145: not primary signals.
ORDET (Italy) – Muti et al., 2014	Nested case–control; 133 cases / 133 controls	miRNA profiling in leukocytes (platform per original publication)	20 miRNAs differentially expressed; 15 down-regulated. miR-145-3p and miR-145-5p consistently down-regulated in cases.	miR-145-3p/5p: lower in cases. miR-181 and miR-222: not primary signals.
